# Control of Avian Coccidiosis: Future and Present Natural Alternatives

**DOI:** 10.1155/2015/430610

**Published:** 2015-02-17

**Authors:** Rosa Estela Quiroz-Castañeda, Edgar Dantán-González

**Affiliations:** Laboratorio de Investigaciones Ambientales, Centro de Investigación en Biotecnología, Universidad Autónoma del Estado de Morelos, 62210 Cuernavaca, MOR, Mexico

## Abstract

Numerous efforts to date have been implemented in the control of avian coccidiosis caused by the *Eimeria* parasite. Since the appearance of anticoccidial chemical compounds, the search for new alternatives continues. Today, no product is available to cope with the disease; however, the number of products commercially available is constantly increasing. In this review, we focus on natural products and their anticoccidial activity. This group comprises fatty acids, antioxidants, fungal and herbal extracts, and immune response modulators with proven anticoccidial activity, many of which exist as dietary supplements. Additionally, we offer an overview of the poultry industry and the economic cost of coccidiosis as well as the classical strategies used to control the disease.

## 1. Introduction

Worldwide, the poultry industry spends a significant amount of money in the prevention and treatment of several diseases. One of those diseases, avian coccidiosis, is caused by several species of the protozoan parasite* Eimeria*. This parasite invades epithelial tissues of the intestine, causing severe damage in birds and as a result, significant economic losses. The main problem with* Eimeria* infections is that they are caused by more than one species that attack different regions of the intestine. The use of several drugs, alone or in combination, has proven to be an effective alternative in the struggle against avian coccidiosis. However, the emergence of drug-resistant strains, especially after a prolonged use of a drug, is a real problem. Thus, vaccines are the only preventative methods. Due to this, new alternatives have emerged, most of which are natural compounds extracted from plants or produced by microorganisms. Some of these compounds are antioxidants that damage the parasite, thus preventing the infection. Others, such as essential oils, disrupt the structure of oocysts preventing the dispersion of the parasite. From this perspective,* Eimeria* parasites should be controlled by several ways, implying the use of a toolkit yet to be available.

## 2. Overview of the World Poultry Industry

The poultry industry is one of the most important food suppliers in the world. Chicken meat represents an important source of animal proteins and fats, as well as a source of a whole range of organic and inorganic substances [1]. The chicken meat market represents a very dynamically developing area, with a significant increase in production with time. European and American countries dominated the chicken market fifty years ago with 79% of total production. However, by 2012, Asian and American countries contributed approximately 77% of total world production [2]. In the period between 2000 and 2012, world chicken meat production increased by 58.48%. The increase differed according to geographical location and was as follows: Asian production increased by 68.83%, production by Australia and New Zealand increased by 68.49%, African production increased by 67.73%, European production increased by 65.82%, and production by the Americas increased by 47.67%. Currently, world production is dominated by the USA, China, Brazil, Mexico, Russia, and India [2].

This growing demand of meat is related to the increasing number of inhabitants in the world and their increasing purchasing power, in addition to the fact that chicken meat is cheaper than other types of meat [3]. Although the supply of meat in the world has increased, factors that limit the progress of this industry still exist, such as the handling, housing, and rearing of birds in addition to disease control (nutritional, metabolic, and parasitic diseases).

In an industry that raises approximately 40 billion chickens annually, avian coccidiosis represents a serious disease that results in annual global economic losses of approximately $2.4 billion, including production losses and disease prevention and treatment costs [4–6]. This disease is a major cause of mortality, poor performance, and lost productivity in domestic livestock, mostly because modern production involves the rearing of large numbers of birds in small confinement areas at high densities, thus assisting in parasite dispersal. For example, in a modern broiler house, 200–500 birds are housed under one roof at a stocking density of one bird per m^2^ [7].

The ability of coccidiosis-causing parasites to survive environmental challenges as well as the emergence of drug resistance represents a serious threat to the secure production of poultry-derived food products [6].

## 3. Causative Agents of Coccidiosis

Parasites causing coccidiosis are commonly found in places where chickens are raised. The most common means of coccidian parasites spreading is related to the movement of personnel between houses and farms. Coccidiosis is a self-limiting disease, and its manifestation depends on the number of oocysts ingested and the immune status of the host bird [8].

Avian coccidiosisis caused by several species of* Eimeria* (family Eimeriidae) that belong to the phylum Apicomplexa. This phylum comprises many members of entirely parasitic diseases of humans and animals with a wide environmental distribution. Organisms belonging to this phylum are obligate intracellular parasites characterized by unique specialized organelles, most notably those from within the apical complex (micronemes, rhoptries, dense granules, and conoid and polar rings), that would provide the structural stability required during the host invasion process [9, 10]. More than 5000 species of apicomplexan parasites have been reported, including* Plasmodium falciparum*, the causative agent of malaria;* Babesia* and* Theileria*, cattle parasites;* Cryptosporidium*,* Toxoplasma*, and* Sarcocystis*, human and animal pathogens; and* Eimeria* sp., poultry and cattle pathogens [10]. The damage caused by these parasites is host-specific and is based on the uncontrolled cycles of host cell invasion, parasite proliferation, host-lysis, and reinvasion.

Over 1000 species of* Eimeria* are known, and most parasitize the intestinal epithelia of vertebrates including those of horses, domestic dogs and cats, rabbits, cattle, sheep, pigs, turkeys, and chickens, with an economically significant burden [11]. In the domestic fowl (*Gallus gallus*), nine different* Eimeria* spp. are described [12, 13].

*Eimeria* invade and destroy the intestinal epithelium of chickens, and as a consequence, infected birds display reduced feed intake, have bloody diarrhea, and have hampered weight gains [14, 15].

Infections are not caused by a single species of* Eimeria*. Instead, the disease can be considered to come as a result of a mixture of* Eimeria* spp. In fact, this parasite develops in different regions of the gut and depending on the magnitude of the infection; they can cause mild-to-severe lesions.

Each species of parasite has a predilection for a specific site in the gastrointestinal tract. For instance,* E. acervulina* develops in the duodenum,* E. maxima* and* E. mitis* develop in the middle part of the small intestine,* E. tenella* develops in the caeca,* E. brunetti* develops in the caeca and the rectum, and* E. necatrix* develops in the small intestine [16].

The species of* Eimeria* that are reported as highly pathogenic are* E. brunetti, E. maxima, E. necatrix,* and* E. tenella.* Species reported as mildly pathogenic include* E. acervulina, E. mitis,* and* E. mivati*, whereas* E. praecox* and* E. hagani* are considered to be the least pathogenic [17, 18] (Table 1).

## 4. The Life Cycle of* Eimeria*

The life cycles of* Eimeria* spp. are complex, consisting of two developmental stages in the host: an exogenous stage (sporogony) and an endogenous stage (schizogony and gametogony). Some species vary in the number of asexual generations and in the time required for each developmental stage [6, 19].

During the exogenous phase, the unsporulated (noninfective) oocyst is excreted from the chicken and undergoes sporulation in the presence of moisture, warmth, and oxygen, thus becoming a sporulated (infective) oocyst. Sporulated oocysts of* Eimeria* contain four sporocysts, each containing two sporozoites. The endogenous phase occurs in the intestine of the host and involves several rounds of asexual reproduction (schizogony) followed by sexual differentiation (gametogony), fertilization, and the shedding of an unsporulated oocysts [4, 20].

Al least two generations of asexual development (sometimes as many as four) give rise to a sexual phase, where small, motile microgametes seek out macrogametes to form the zygote which matures into an oocyst that is released from the intestinal mucosa and is ultimately shed in the feces. Thus, the reproductive potential of a single ingested oocyst is fairly constant [19].

The short life cycle (4–6 days, depending on the species) and the copious production of sporulating oocysts are advantageous for increasing the chances of infecting a large population of chickens.

Infection occurs when the host ingests sporulated oocysts. Following ingestion, the microenvironment of the host digestive tract stimulates excystation of the oocyst in the gizzards resulting in the release of sporozoites that invade and destroy cells in the intestinal mucosa and begin the reproductive cell cycle. As a consequence, infected birds display symptoms of disease such as reduced feed intake, bloody diarrhea, and hampered weight gain [14, 15].

## 5. The Coccidian Oocyst

The oocyst is considered a remarkably hard and persistent structure. It is resistant to mechanical and chemical damage and to proteolytic degradation [21, 22]. In fact, oocysts remain viable and infectious after treatment with diluted sodium hypochlorite or after storage with the strong oxidizing agent potassium dichromate (1 to 2%) or with sulfuric acid. Actually, each layer of the oocyst wall responds very differently to chemical treatments [21, 23, 24]. The oocyst wall is a structure that is composed of a bilayer, with an outer layer that can be as thick as 500 to 600 nm but that is eventually compacted to 200 nm or less. An inner zone of approximately 40 nm separates the outer and inner layers, with the latter being approximately 40 nm thick [25]. The oocyst is noninfective when being in its unsporulated state and becomes infective when it sporulates [4, 20]. It has been found that an unsporulated oocyst can survive up to seven months in the cecal tissue and that the sporulated oocyst can survive up to 602 days in the exogenous environment [26].

Basically, the composition of the two walls confers the oocyst with an outstanding resistance. The oocyst wall consists mainly of proteins and lipids. In addition, carbohydrates covalently bonded to proteins have also been reported, with varying percentages (1.5–19%). However, the composition of each layer is still unclear [21, 27].

The inner wall is composed of a protein matrix embedded with as well as coated with lipids, while the outer wall is composed of quinone tanned proteins as well as protein-tyrosine crosslinks. Although the presence of cysteines has been reported, no cysteine bridges have been detected [28, 29].

The proteins provide the oocyst with great structural stability against extreme heat or cold because the oocysts are sensitive to high temperatures and desiccation. It is likely that the outer layer protects the oocyst from mechanical damage whereas the lipid-rich inner layer protects it from chemical attack [21, 28].

The oocyst is a defining characteristic of the coccidian; they are the result of the fusion of micro- and macrogametes and are usually shed with feces. In most cases, the oocyst is unsporulated and is considered as noninfectious when excreted and contains a single, undifferentiated cytoplasmic mass protected by a wall. Only under favorable environmental conditions of humidity, temperature, and oxygen at the right amounts make the sporulation occur [30]. During this process, the cytoplasm divides into secondary sporoblasts, which develop a resistant wall and are then called sporocysts. Within the sporocysts, the sporozoites are formed and are considered as the infectious stages. The number of sporocysts varies between species of coccidians. For instance, in* Eimeria* there are four sporocysts, each with two sporozoites, while* Isospora* and* Toxoplasma* have two sporocysts with four sporozoites each [21, 31].

## 6. Strategies to Control Avian Coccidiosis

Although good husbandry can help in reducing the risk of transmission of coccidiosis-causing parasites, additional measures are essential to accomplish a complete control of the disease [5]. Some efforts have focused on the development of anticoccidial compounds that attack both the sexual and asexual stages of the parasites (stages that occur within the host) rather than targeting the most infectious stage, the oocyst [32].

The agents used for the prevention and control of coccidian infections are termed anticoccidial drugs. These drugs can either be coccidiostatic or coccidiocidal agents. The former comprises drugs that prevent the replication and growth of coccidial populations, whereas the latter includes drugs that destroy coccidial populations. In general, coccidiocidal drugs have been more effective than coccidiostatic drugs. This has been the case because when coccidiostatic medication is withdrawn, arrested parasites may continue their life cycle and go on to contaminate the environment with infective oocysts [8].

Two categories of drugs are employed in the poultry industry, those being ionophorous compounds (ionophores) and synthetic drugs (chemicals). Generally, ionophores cause the death of the parasite by interfering with the passage of ions across the cell membrane, whereas chemicals act by inhibiting different biochemical pathways of the parasite [32].

Synthetic drugs were the first to be discovered and comprise a diverse array of molecules that are absorbed into the blood stream of the host and kill developing parasites in the epithelial cells of the villi in the intestines. One of the oldest synthetic drugs is nicarbazin (coccidiostatic agent). The molecular mechanism of nicarbazin is based on inhibiting the development of the first and second generations of the schizont stage of the parasites. There are some molecular mechanisms proposed for nicarbazin's avian adverse effect, but no research group to date has conducted* in vivo* research [33]. Nicarbazin is one of the most successful drugs and is still widely used today [27, 34]. Another coccidiostatic synthetic drug with a wide range of action is amprolium and has been shown to inhibit the uptake of thiamine by second generation schizonts of* E. tenella*. Quinolone drugs inhibit cellular respiration by blocking the electron transport chain in the parasite mitochondrion thus arresting the parasite in the early stages of development [35, 36]. Ionophores, which are by-products of bacterial fermentation, have a unique mechanism of action. They are known to affect cellular processes involving cation (mono and divalent) transport through the cell membrane thus affecting the osmotic balance [7, 11]. These compounds are considered as coccidiocidal because of their ability to preferentially move ions, usually sodium, which results in highly toxic conditions to the cell [19]. Besides properties mentioned, ionophores show a broad spectrum of bioactivity ranging from antibacterial, antifungal, antiparasitic, antiviral, and tumor cell cytotoxicity [37, 38].

Since the discovery of sulfonamide sixty-five years ago as a potent compound to control* Eimeria* infections, the development of anticoccidial drugs has continued in earnest. The use of several drugs, alone or in combination, has proven to be an effective mechanism in the struggle against avian coccidiosis. However, the emergence of drug-resistant strains, especially after prolonged uses of a drug, is a real problem [8].

To combat resistance, shuttle and rotation systems of drugs are employed. In the shuttle program, the different drugs are used during a period of juvenile growth to market-size growth, whereas in the rotation program, the type of drug used is switched after one or several grow-out periods or seasonally [39].

However, even with the shuttle and rotation programs there is no method to fully prevent drug resistance. This has been observed when ionophores, such as monensin or lasalocid, are used in the field and drug resistant parasites emerge [40].

Due to the constant pressures by government agencies and consumers to ban the use of drugs in animals intended for human consumption, other alternatives to the control of coccidiosis are now available. The demand for alternative methods has constantly increased in European countries, Australia, and the US [41–43]. Consequently, the development and use of vaccines and other alternatives have showed a significant increase.

Immunity to* Eimeria* is stimulated by the initial developing parasite stages, particularly the schizonts, and is subsequently boosted and maintained by multiple reexposures to oocysts in the litter. Accordingly, the recycling of infection, following the administration of live oocysts, is critical for the development of protective immunity [44].

Two types of vaccines are currently used with the aim of controlling coccidiosis in a chemical-free way: unattenuated and attenuated vaccines. Their effectiveness is based on the recycling of what are initially very low doses of oocyst and on the gradual buildup of solid immunity [45].

The use of live unattenuated vaccines (Coccivac, Advent, Immucox, and Inovocox) is limited due to the risk induced by the live parasites, so their use is accompanied by chemical treatments to control the inherent pathogenicity of the parasites. However, this practice is no longer required due to the improved methods of administration of the oocysts [46].

The success of live attenuated vaccines (Paracox and HatchPak CocciIII) is based on the fact that there is a lower risk of disease occurring because there is a reduction in the proliferation of the parasites and as a result less damage to the intestine of the bird [41]. Today, attenuation of* Eimeria* species is based on precociousness. This refers to populations of parasites that complete their life cycle up to 30 h faster than parasites from the same parent strain, resulting in parasites with attenuated virulence and a significant reduction in their reproductive capacity [5, 47, 48]. Today, precocious lines are described for all species of* Eimeria* [42].

Although the species-specific nature of immunity induced by exposure to live* Eimeria* (whether attenuated or un-attenuated) is significant to control coccidiosis, there still is a requirement for the development of a fully effective anticoccidial vaccine, mainly because* Eimeria* species distribution can vary between poultry farms and specific screenings should be performed before vaccine administration.

Additionally, the anticoccidial drug resistance observed in birds around the world has directed the search for natural products with efficient anticoccidial activity [49]. For more detailed information about vaccines, the follow published reviews are good resources [7, 50].

## 7. Natural Alternatives to Controlling Coccidiosis

There is a current interest in the use of so-called natural products, which include fungal extracts, plant extracts, and probiotics to reduce problems caused by coccidiosis [7].

Many of these natural compounds are used as diet supplements with varying effects that include immune stimulation, anti-inflammatory and antioxidant activities, and cytoplasmic damage [51].

### 7.1. Fats

It has been reported that sources of fat containing high concentrations of docosahexaenoic acid, eicosapentaenoic acid, and linolenic acid (known as n-3 fatty acids) from fish oils or flax seeds reduced the severity of* Eimeria tenella* infections in young broiler chicks. Diets supplemented with 2.5 to 10% fish oil, 10% flax seed oil, or 10% linseed oil significantly decreased cecal lesions, which allowed a maintained weight gain in birds. In addition, a reduced parasite invasion rate and development were also observed in the caeca of infected chicks. Unfortunately, the effect of these fatty acids was only observed in* E. tenella* infected animals but not in* E. maxima* infected animals [52, 53]. In fact, diets with low levels of linolenic acid do not show protection against* E. tenella* infection [54].

These results suggest that these diets induce an oxidative stress that is detrimental to parasite development, and this may be because sporulated oocysts and sporozoites of* E. tenella* are deficient in superoxide dismutase, an enzyme that would protect them from reactive oxygen species [55].

### 7.2. Antioxidants

Cells are constantly exposed to environmental damage or to damage caused by the cells themselves. In response to this damage, antioxidant molecules are important to control and reduce oxidative stress caused by increased levels of reactive oxygen species and free radicals that can initiate chain reactions in the cell, resulting in the death or in serious damage to the cell [56]. In the poultry industry, the use of antioxidants from natural sources can help in restoring the balance of oxidants/antioxidants, leading to an improvement of birds infected with coccidiosis. Fruits and other plant materials provide a good source of an antioxidant due to their high content of phenolic compounds [57].

Most of the antioxidants available are found as dietary supplements. One of the most studied antioxidant is vitamin E, known to delay lipid peroxidation in muscles and improve meat quality. Various fruit and herb plants such as plum, cranberries, pomegranate, bearberry, grape seed extract, pine bark extract, rosemary, oregano, green tea, and other spices function as antioxidants in meat and poultry products [57–59]. Curcumin, present in* Curcuma longa*, could reduce the severity of an infection of the upper and middle part of the small intestine caused by* E. acervulina* and* E. maxima* [60].

Naidoo et al. [61] used the antioxidants properties of several plant extracts and compared those to the drug toltrazuril. They reported that* Tulbaghia violacea*,* Vitis vinifera*, and* Artemisia afra*, used in doses of 35 g/kg, 75 mg/kg, and 150 mg/kg, respectively, exhibit an activity similar to that observed in the control drug.

Artemisinin, an extract isolated from* Artemisia annua*, is effective in reducing oocyst shedding output from* E. acervulina* and* E. tenella* but not* E. maxima* infections when broiler chickens are fed the extract at concentrations of 1 or 2.5 mg/kg. The extract's mechanism of action is thought to involve oxidative stress [62].

### 7.3. Essential Oils

Most of the alternative therapies offered in the treatment of coccidiosis are focused on attacking the stages of the parasite that are different from the oocyst.

The use of essential oils as part of formulations or diets to control coccidiosis has been reported. Recently,* in vitro* destruction of* Eimeria* oocysts was reported after a three hours contact period with essential oils from artemisia, thyme, tea tree, and clove [63]. Out of ten essential oils tested, only those four present with a LC_50_ < 1 mg/mL for oocysts.

Oocysticidal activity of the commercial oils carvacrol, carvone, isopulegol, thymol, and eugenol was also evaluated. The lysis was monitored in suspensions of oocysts from* E. tenella* (45%),* E. maxima* (32%),* E. acervulina* (10%),* E. necatrix* (6%), and* E. mitis* (7%) through the release of internal substances at 273 nm [64]. Although the mechanism of action of essential oils is still unknown, these two reports are an example of the use of natural substances as agents for the destruction of the most resistant structure of the parasite, the oocyst. Nevertheless, the economic factor for obtaining these products could be an impediment for their extensive use in farms.

### 7.4. Herbal Extracts and Medicinal Plants

Extracts from plants have also been shown to exhibit anticoccidial effects. Youn and Noh [65] assessed the effect of 15 different herbs against* E. tenella* in one-day old broiler chickens. They found that survival rates in the groups treated with* Ulmus macrocarpa*,* Pulsatilla koreana*,* Torilis japonica*,* Artemisia asiatica*, and* Sophora flavescens* were higher than those of the infected control. Bloody diarrhea in the* S. flavescens* and* Sinomenium acutum*-treated groups was milder when compared to the control-treated groups. Lesion scores in the groups treated with* U. macrocarpa* and* P. koreana* were significantly lower than those of the control group. In summary, the data of survival rates, bloody diarrhea symptoms, lesion scores, body weight gains, and oocyst excretions indicate that* S. flavescens* was the most effective, followed by* P. koreana*,* Sinomenium acutum*,* U. macrocarpa*, and* Quisqualis indica*.

A comparison between* Artemisia sieberi* extracts and ionophore monensin was assessed to compare their anticoccidial effect on 21-day old broiler chickens infected with* E. tenella*,* E. maxima*,* E. necatrix*, and* E. acervulina* [66]. This study showed that chickens challenged by coccidiosis and treated with* A. sieberi* extract had a decreased number of oocysts per gram of feces and had improved growth performance parameters such as feed intake and weight gain, among others, when compared with the effects observed with monensin treatment. The extract could be an alternative therapeutic agent against avian coccidiosis under field conditions.

Similar results were observed with an extract of* A. sieberi* obtained using petroleum ether and recovered as a novel granulated extract. One-day old broilers challenged by* E. tenella* on day 21 and treated with this extract showed a significant reduction in mortality, diarrhea, lesion scores, and oocyst number in feces. The authors suggest that this new formulation is a promising herbal medicine that can be used as a prophylactic or therapeutic product to control avian coccidiosis [67].

The extraction of compounds from herbal material is common. Recently, Ola-Fadunsin and Ademola [68] used* Moringa oleifera* acetone extract and assessed its anticoccidial activity. They used this extract to treat broiler chickens naturally infected with several* Eimeria* species. The parameters assessed were inhibition of oocyst output, fecal score, weight gain, and mortality, and in all cases tested, positive results were obtained. Additionally, evaluation of hematological indices showed a significant increase in packed cell volume, hemoglobin concentration, and red blood cell count of the treated birds.

Some plants used against* Eimeria* also possess activities against other protozoan parasites such as plasmodia and trypanosomes, which makes the plant or its extracts a feasible phytomedicine [69]. For plants such as* Eclipta alba*, other biological activities, in addition to anticoccidial activities, are reported, such as antimicrobial, analgesic, antiviral, anti-inflammatory, and others [70].

Different diet supplementation with plant-derived phytonutrients, carvacrol, cinnamaldehyde, and capsicum oleoresin has been used to examine their immunomodulatory effects on broiler chickens infected with* E. acervulina* [71]. The results of this study provide evidence that these phytonutrients possess immune enhancing properties in chickens, which offers the possibility of developing effective drug-free alternative strategies to control poultry coccidiosis [71] (Table 2).

### 7.5. Immune Response Modulators

Enhanced immune responses were observed in one-day old chickens fed with a lyophilized powder extracted from plums. These chickens show an increased body weight gain, a reduced fecal oocyst shedding rate, and an increase in the mRNAs for IFN-*γ* and IL-15. Furthermore, chickens fed with plum exhibited a greater spleen cell proliferation [72].

In this search for natural products, the use of probiotics and prebiotics emerges as an alternative to the use of antibiotics on a large scale. The use of these products have been shown to prevent the establishment of pathogens in the intestinal tract of chickens, thus increasing weight gain, feed conversion ratio, and livability, in addition to acting as immune response modulators [73, 74].

Probiotics are defined as live microbial feed supplements designed to benefit the host by improving the intestinal microbial ecology [75]. The commercial probiotic MitoMax, containing* Pediococcus acidilactici* and* Saccharomyces boulardii*, was evaluated as an alternative control method to prophylactic drugs against coccidiosis [74].

Chickens fed with MitoMax, a commercial mixture of fermentation cultures, at concentrations of 0.1%, and challenged with* E. tenella* or* E. acervulina* showed an enhanced humoral immunity and significant changes in body weight gain and fecal oocyst shedding rates.

Diet enriched with lactobacilli has also been used, acting as immunomodulators to stimulate the gut-associated bacteria in neonatal chicks, thereby protecting them from disease without decreasing growth performance. This has been proposed as a possible substitution to antibiotics [76]. Probiotics, in combination with vaccines, were used to observe the immune response in broilers. Stringfellow et al. [77] observed an increase in lymphocyte proliferation on day 14 in addition to higher levels of heterophil oxidative bursts at day 7. These results confirm that probiotic treatments are very useful in modulating the immune response.

In addition to probiotics, a number of nonspecific immunomodulatory agents have been used to enhance immune responses against several pathogens in the poultry industry. For instance, heat-killed* Mycobacterium phlei* exhibited an immunotherapeutic potential in broiler chickens infected with* E. tenella*. Broilers treated with* M. phlei* showed a significant body gain weight and caecal lesion score. Additionally, these bacteria can act as immunostimulant agents and have beneficial roles against caecal coccidiosis [78].

Arabinoxylans derived from wheat (*Triticum aestivum*) have also been shown to have immunostimulatory and protective effects against coccidiosis in broiler chickens [79].

Recently, a number of herbal complexes and botanicals, such as the fungi* Lentinula edodes* and* Tremella* and the plants* Aegle marmelos*,* Eclipta alba*,* Olea europaea*,* Pinus radiata*, and* Echinacea purpurea*, have been shown to contain active ingredients with mechanisms of action that include immune stimulation, whether alone or in combination with vaccines [51, 80].

The combination of plants extracts was also used to evaluate anticoccidial activity, with the aim of obtaining a higher activity. Almeida et al. [81] combined* Artemisia annua* and* Curcuma longa* ethanolic extracts to treat broilers infected with* E. acervulina *and* E. maxima*. The activity of both extracts was compared with broilers treated with chemical coccidiostats as controls. They report that broilers given a supplement of chemical coccidiostats exhibited better protection against* Eimeria* infection. However, when higher doses of the herbal mixture were used, a reduced mortality and reduced numbers of oocysts were observed, as well as protection against lesions, when compared with the positive control. Still, further investigations into dosages and mechanisms of action of this combination should be performed. These results suggest that herbal extracts could be used as supplements of diets in combination with other management practices to reduce the use of synthetic drugs.

Most recently, the efficacy of in-feed preparations including five dietary supplement regimens (anticoccidial salinomycin, probiotic, prebiotic, multienzyme, and essential oils mixture) were evaluated in* Eimeria* spp. infected broilers [82]. Infected animals treated with salinomycin, multienzyme, probiotic, and prebiotic, but not essential oil mixtures, showed a significant improvement in body weight gain and feed conversion ratio, which suggests that these supplements could reduce adverse effects after a challenge with a coccidiosis-causing agent. Moreover, in the infected broilers, all of the supplements reduced the severity of coccidiosis lesions, whereas supplementation with salinomycin or essential oil mixture alone resulted in a significantly reduced oocyst excretion rate.

An interesting example of the use of yeast in inducing the immune response is* Pichia guilliermondii*. The killed whole yeast cell and all its components are commercially available as CitriStim (ADM, Quincy, IL), a product considered as a good source of mannan oligosaccharides and *β*-glucans known for their immunomodulatory effects [83, 84].

This product contains a proprietary mixture of partially fermented yeast that is left following citric acid extraction from the yeast culture. Broilers fed with CitriStim exhibited a decrease in the number of oocysts excreted seven days after coccidial challenge (Inovocox, Pfizer Animal Health, NY) thus accelerating the clearance of the coccidian. In regard to the immune response, the authors observed an increased macrophage nitric oxide production and inflammatory cytokine production postcoccidial infection.

Despite the anticoccidial activity observed for natural products, the elevated cost of farming and the production required to obtain sufficiently large quantities of extracts renders their use as a strategy to control coccidiosis in large population of birds difficult. However, the good results observed through the use of natural alternatives could compensate for this struggle. On the other hand, as many of the extracted products contain chemical compounds, a deep analysis of their toxicity to animals or humans should be performed before any commercial application.

Although many natural products are still under experimental stages, nowadays, a wide range of natural products are commercially available; in Table 3, some of these products are shown.

## 8. Conclusions

Over time, anticoccidial drug development has increased in response to the urgent need to control avian coccidiosis. Today, we have several strategies available, many of which are currently widely used in chicken farms. Moreover, new alternatives are emerging, as is the case with anticoccidials obtained from plants, fungi, or microorganisms. One of the advantages of using natural extracts is the lower risk of developing resistance, such as that observed with chemical drugs. It is widely known that the availability of raw materials and the cost of production could be high in the development of natural extract alternatives. However, the cost is well worth it if we consider that these alternatives are friendly to the environment, producers, and consumers.

## Figures and Tables

**Table 1:** Main characteristics of *Eimeria* species.

Species	Site of development	Pathogenicity	Gross lesions	Reference
*E. praecox *	Duodenum, jejunum	Least pathogenic	Watery intestinal contents Mucus and mucoid casts	[8]
*E. hagani *	Duodenum, jejunum and ileum	Least pathogenic	Petechiae and white opacities in the upper small intestine Intestinal content may be creamy or watery	[19]
*E. acervulina *	Duodenum, ileum	Less pathogenic	Limited enteritis causing fluid loss. Malabsorption of nutrients.	[50]
*E. mitis *	Ileum	Less pathogenic	Limited enteritis causing fluid loss. Malabsorption of nutrients	[50]
*E. mivati *	Duodenum, rectum	Less pathogenic	Red petechiae and round white spots Severe denuding of the mucosa	[50]
*E. maxima *	Jejunum, ileum	Moderately-Highly pathogenic	Inflammation of the intestinal wall with pinpointed hemorrhages Sloughing of epithelia	[50]
*E. brunetti *	Caeca and rectum	Highly pathogenic	Inflammation of the intestinal wall with pinpointed hemorrhages Sloughing of epithelia	[50]
*E. tenella *	Caeca	Highly pathogenic	Thickened cecal wall and bloody contents at the proximal end Distension of caecum Villi destruction causing extensive hemorrhage and death	[50, 85]
*E. necatrix *	Jejunum, ileum, caeca	Highly pathogenic	Intestine may be ballooned Mucosa thickened and the lumen filled with fluid, blood and tissue debris Lesions in dead birds are observable as black and white plaques (salt and pepper appearance)	[50]

**Table 2:** Evaluation of the activity of compounds supplemented in the diets of birds infected by *Eimeria* spp.

Source/compound	Parasite	Oocysts per gram in feces (dpi)	Body weight gain (g)	Lesion^*^	Hematological parameters^*^	Immune response analysis^*^	Reference
*Ageratum conyzoides* (80% ethanol extract)	Et	56 × 10^4^ (18)	900^a^	NE	E	NE	[69]
*Eclipta alba* (coumestans from methanolic extraction)	Et	4.67 (14)	370	E	NE	NE	[70]
Tannic acid	Ea, Em, Et	3400 (23)	NE	E	NE	NE	[86]
*Curcuma longa*/*Capsicum*/ *Lentinula edodes *	Ea	4.6 × 10^7^ (10)	550^a^	NE	NE	NE	[87]
Carvacrol/ Cinnamaldehyde/ Capsicum oleoresin	Ea	4.5 × 10^7^ (9)	700^a^	NE	NE	E	[71]
Garlic (Propyl thiosulfinate/Propyl thiosulfinate oxide)	Ea	1.3 × 10^8^ (6–9)	570^a^	NE	NE	E	[88]
*Emblica officinalis* (tannins)	Ea, Em, En, Et	50 × 10^3^ (12)	12^a,b^	E	NE	E	[89]
*Musa paradisiaca* (methanolic extract)	Et	0.45 × 10^3^ (7)	NE	NE	E	NE	[90]
*Ganoderma lucidum* (aqueous extract)	Et	0 (7)	E	NE	E	NE	[91]
*Aloe vera *	Em	1.6 × 10^7^ (6–10)	260^a^	E	NE	NE	[92]
Green tea	Em	19 × 10^6^ (7)	60^a^	NE	NE	NE	[58]

^a^Approximated value; ^b^dry weight gain; dpi: days post-infection; E: parameter evaluated; NE: parameter not evaluated; Et: *E. tenella, *Em*: E. maxima,* Ea: *E. acervulina, *and En:* E. necatrix*. ^*^Descriptive characteristics.

**Table 3:** Some natural products commercially available for prevention and treatment of coccidiosis.

Product	Ingredients	Supplier
Avihicox	Clove and *Bocconia cordata* extract	Centaur
Nutrimin	Apple cider vinegar	Chicken Lickin
Kocci Free	Olive leaf, mustard seed, black seed, cloves, grapefruit seed extract.	Amber Technology
Oil of oregan	Oregan extra virgin olive oil (80% carvacrol)	Natural factors
Oilis	Natural vegetal extracts	Engormix
Oreganico	Oregan oil and essential oils	Flyte so fancy
Garlic granules	Garlic	Flyte so fancy
Poultry Provita	Probiotics and prebiotic inulin	Vets Plus
CitriStim	Mannan oligosaccharides and beta glucans	ADM
Orego Stim	Carvacrol (82%) and Thymol (2.4%)	SaifeVetmed
Herban	Etheric oils, soya oils, oregan oils	Uncle Ted Organics Ltd
Herb 'n' thrive	Concentrated blend of herbs and essential oils	Chicken Lickin
Eimericox	Several essential oils	Phytosynthese/Trouw Nutrition
Natustat	Several essential oils and yeast cell walls	Alltech
Enteroguard	Garlic and cinnamon	Orffa
Xtract Immunocox	Spanish pepper and turmeric	Pancosma
Coxynil	*Allium sativum* Linn 15%, *Cinnamonum camphora* Nees & Eberum 15%, *Elephantopus scaber* Linn 15%, *Valeriana wallicgii* DC 15%, Sulphur dioxide 25% and NaCl 15%.	Growell India
Ropadiar Solution	Oregan oil (on diatomite)	Ropapharm
